# On the Poisonous Effects of Meadow Saffron in Cattle

**Published:** 1814-09

**Authors:** J. Want


*For the Medical and Physical Journal.*

### *On the poisonous Effects of Meadow Saffron in Cattle.*

A STATEMENT has appeared in one of the morning papers, of some yearlings having been killed by the eating of colchicum. This account is evidently a fabrication, as at this season of the year no part of the plant is above ground, and, consequently, not within their reach. I am indebted for this fact to Sir Joseph Banks, and Mr. Andrew Knight, the president of the Horticultural Society; and to the latter of these gentlemen for some particulars on this subject, which are of sufficient importance to communicate. Cattle are affected by the meadow saffron *only* at the spring of the year, when the seed-vessel is fully mature. It appears that the seed, if swallowed, adheres to the coat of the stomach, producing at the several points of its adhesion spots of inflammation, which occasion the death of the beast. It is a curious fact, that they are affected by the recent plant *only*; for, when dried and made into hay, it loses its deleterious property, and is then eaten by them with impunity. Whether, if taken in great quantity, it might not still be poisonous, has not, I believe, been determined. I am disposed to account for the peculiar effect of the seed, from its property of adhering to the coat of the stomach; this *may* be lost in the dried state, by which it is enabled to mix with, and is diluted by, the food. J. WANT.

